# The non-linear association between preoperative platelet to white blood cell ratio and 30-day postoperative mortality in adult tumor craniotomy: a retrospective cohort study

**DOI:** 10.3389/fneur.2026.1852566

**Published:** 2026-07-20

**Authors:** Jiangheng Guan, Lei Yu, Xiuwen Hu, Shaomin Chen, Jian Wang, Li Tan

**Affiliations:** 1 Department of Neurosurgery, General Hospital of Central Theater Command, Wuhan, China; 2 Department of Disease Control and Prevention, General Hospital of Central Theater Command, Wuhan, China; 3 Chinese PLA Center for Disease Control and Prevention, Beijing, China

**Keywords:** 30-day mortality, brain tumor, craniotomy, non-linear, platelet to white blood cell ratio

## Abstract

**Objective:**

While the platelet to white blood cell ratio (PWR) has been linked to mortality risk in diverse clinical settings, evidence regarding this association in tumor-specific populations, particularly following intracranial tumor resection, is lacking. This study aimed to investigate the association and explore the specific non-linear relationship between preoperative PWR and 30-day postoperative mortality in adult patients undergoing intracranial tumor craniotomy.

**Methods:**

This retrospective cohort study conducted a secondary analysis of data from 9,434 patients who underwent craniotomy for intracranial tumors, utilizing the ACS NSQIP database. The exposure variable was preoperative PWR, while the outcome of interest was 30-day postoperative mortality. Cox proportional hazards models were employed to investigate the association between them. To explore non-linear relationships, an additive Cox proportional hazards model incorporating smooth curve fitting was employed, and a two-piecewise Cox regression model was utilized to assess the threshold effect.

**Results:**

The study population exhibited a mean preoperative PWR of 27.044 ± 12.564, with a 30-day postoperative mortality rate of 3.148% (297/9434). Multivariable analysis revealed that preoperative PWR was inversely associated with 30-day postoperative mortality (HR = 0.978, 95% CI: 0.967–0.990). Furthermore, a non-linear association between them was observed, with a PWR inflection point at 20.111. Below this value, each unit increase in preoperative PWR was associated with an approximate 8% decrease in the risk of 30-day mortality (HR = 0.920, 95% CI: 0.892–0.949). However, the association was not significant above it (HR = 0.997, 95% CI: 0.984–1.011). Sensitivity analyses confirmed the robustness of our findings.

**Conclusion:**

Our study demonstrated an inverse and non-linear association between preoperative PWR and 30-day postoperative mortality in U.S. adults undergoing intracranial tumor craniotomy. These findings warrant confirmation in future studies.

## Introduction

Craniotomy-based surgical resection constitutes a principal therapeutic strategy for the management of intracranial tumors in adults (1). However, this invasive procedure is inherently associated with substantial perioperative complications and risks (2). The 30-day postoperative mortality is a widely recognized metric for assessing surgical safety and short-term patient outcomes (3). The incidence of this adverse event, while variable across different patient cohorts (4–6), remains a major clinical challenge in the neuro-oncological field. Consequently, investigating preoperative factors linked to this adverse outcome is crucial for enhancing the understanding of short-term surgical outcomes.

Platelets and white blood cells play an integral role in systemic inflammatory and thrombo-inflammatory responses in clinical disorders (7–9). The platelet-to-white blood cell ratio (PWR) is a simple, readily available measure calculated as the platelet count divided by the white blood cell count, and it captures the balance between these two cell lines (10). Importantly, as a composite ratio, PWR integrates platelet-mediated hemostatic function and leukocyte-mediated inflammatory activity into a single measure, offering a highly integrated assessment of thrombo-inflammatory status (11, 12). Evidence to date indicates that PWR is negatively associated with short-term mortality across several clinical contexts, including critically ill patients with atherosclerotic cardiovascular disease and hospitalized patients with acute decompensated heart failure (10, 13). Moreover, a non-linear relationship between PWR and mortality risk has been reported in these settings, suggesting that the inverse association of PWR may be most pronounced at lower PWR levels (10, 13). Nevertheless, evidence in tumor-specific surgical populations is absent. Adult patients with intracranial tumors present a distinctly complex pathophysiological milieu. This population is pathophysiologically distinct: intracranial tumor craniotomy triggers profound systemic inflammatory and hemostatic cascades (14, 15), while the tumor microenvironment itself alters platelet activation, leukocyte mobilization, and thrombo-inflammatory responses (16–18). Whether preoperative PWR is independently associated with 30-day postoperative mortality in this surgical population, and whether such an association follows a non-linear pattern, therefore warrants dedicated investigation.

Building upon this rationale, we hypothesized that in adult patients undergoing intracranial tumor craniotomy, lower preoperative PWR levels would be associated with an elevated risk of 30-day postoperative mortality, potentially in a non-linear fashion. To evaluate this association and investigate whether the relationship follows a linear or non-linear pattern, we conducted a multi-center retrospective cohort study utilizing data from the American College of Surgeons National Surgical Quality Improvement Program (ACS NSQIP) database (2012–2015).

## Methods

### Dataset source

The clinical data for this retrospective analysis were sourced from a publicly available supplementary file accompanying a prior publication (19). Although the foundational data for that study originated from the ACS NSQIP registries spanning 2012 to 2015, the present investigation exclusively utilized the specific cohort prepared and made accessible by the original authors. This dataset comprises the clinical profiles of 18,642 adults who underwent craniotomy for intracranial tumors across approximately 400 medical facilities in the United States. Moreover, as these supplementary materials were disseminated under a Creative Commons Attribution License, which expressly allows secondary data use and open-access reuse with proper citation, our study fully adheres to all pertinent data-sharing and copyright regulations.

### Study population

**Figure 1:**
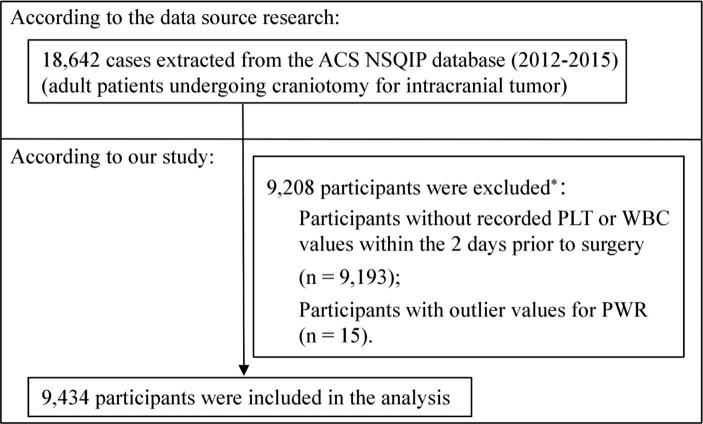
Flowchart depicting the procedural steps for the exclusion of participants. ^*^Exclusion criteria were applied sequentially. ACS NSQIP, American College of Surgeons National Surgical Quality Improvement Program; PLT, platelet; PWR, platelet to white blood cell ratio; WBC, white blood cell.

In accordance with the inclusion criteria established in the original study, the initial dataset of 18,642 adults who underwent craniotomy for intracranial tumors between 2012 and 2015 was assembled using specific Current Procedural Terminology codes: 61510, 61,512, 61,518, 61,519, 61,520, 61,521, 61,526, 61,545, 61,546, and 61,575 (refer to Supplementary Table S1 for detailed information) (19). To ensure accurate assessment of preoperative PWR, participants were required to have recorded platelet (PLT) and white blood cell (WBC) values within 2 days prior to surgery (20). This led to the exclusion of 9,193 individuals. Subsequently, an additional 15 patients with outlier PWR values were excluded. Through this selection process, a final analytical cohort of 9,434 subjects was established (Figure 1).

### Definition of exposure and outcome

The exposure of interest in current study was preoperative PWR. This ratio was computed by dividing the PLT count by the WBC count, with both parameters expressed in units of 10^9^/L.

The outcome of interest was 30-day postoperative mortality, defined as death from any cause occurring within 30 days following the craniotomy procedure. This outcome was determined through the specific data field “days from operation to death” within the ACS NSQIP database (21). Consistent with the database’s standardized protocol, all patients are monitored for the entire 30-day postoperative period to ascertain their vital status, irrespective of hospital discharge status or length of stay. For the purposes of survival analysis, the time-to-event interval was calculated as the number of days from surgery to death for patients who did not survive, whereas those who were alive at the conclusion of the follow-up period were assigned a duration of 30 days.

### Other covariates

Covariates for inclusion were determined *a priori*, guided by clinical considerations and established associations within the existing literature (22–24). The extracted baseline demographics encompassed age range (categorized as 18–40, 41–60, 61–80, and ≥81 years), sex, and race (classified as White, Black, or Other). Furthermore, the analytical models incorporated an extensive array of comorbidities and clinical parameters. These included severe chronic obstructive pulmonary disease (COPD), hypertension, and diabetes. Systemic infection was also accounted for, defined as a binary variable indicating systemic inflammatory response syndrome (SIRS), sepsis, or septic shock. Additional factors considered were open wound infection, disseminated cancer, bleeding disorders, steroid use, and emergency case status. The covariate list further comprised recent weight loss (defined as a loss exceeding 10% of body weight within the 6 months prior to surgery), functional health status (independent vs. dependent), ventilator (denoting required ventilator-assisted respiration at any point within the 48 h prior to surgery), American Society of Anesthesiologists (ASA) classification (categorized as Class I-II vs. Class III-V), along with preoperative blood urea nitrogen (BUN), creatinine, sodium, and hematocrit (HCT).

### Statistical analysis

Continuous variables are summarized as means with standard deviations (SDs) or medians with interquartile ranges (IQRs), depending on their data distribution. Categorical variables are reported as frequencies and proportions. Differences across PWR quartiles were assessed using one-way analysis of variance (ANOVA) or the Kruskal-Wallis test for continuous data, as appropriate, and chi-squared tests for categorical variables.

To assess the association between preoperative PWR and 30-day postoperative mortality, univariate and multivariable Cox proportional hazards regression models were applied. The findings are expressed as hazard ratios (HRs) alongside their corresponding 95% confidence intervals (CIs). The selection of covariates for model adjustment was driven by their clinical significance, potential association with the outcome (22–24), or their ability to alter the main effect estimate by more than 10% (25). Accordingly, the final adjusted models incorporated age range, sex, race, severe COPD, hypertension, diabetes, systemic infection, open wound infection, disseminated cancer, bleeding disorders, steroid use, emergency case, recent weight loss, functional health status, ventilator, ASA classification, BUN, creatinine, sodium, and HCT. Furthermore, the proportional hazards (PH) assumption was evaluated using the Schoenfeld residuals test, yielding a *p* value of 0.0299, which revealed a violation of this assumption. We constructed Kaplan–Meier curves to graphically represent survival probabilities over the follow-up period, comparing the PWR quartiles via the log-rank test.

To investigate potential non-linear relationships between preoperative PWR and 30-day postoperative mortality, we employed an additive Cox proportional hazards model incorporating smooth curve fitting. Subsequently, we utilized a two-piecewise Cox regression model to assess the threshold effect of preoperative PWR on the outcome. Additionally, we conducted a log-likelihood ratio test (LRT) to compare the fit of the one-line Cox model with that of the two-piecewise model, as detailed in previous literature (26, 27).

A series of sensitivity analyses were conducted to evaluate the robustness of our primary findings. First, to address potential population heterogeneity, we performed exploratory stratified analyses across various subgroups using Cox proportional hazards regression models. The significance of interactions was assessed via likelihood ratio tests, comparing models with and without the interaction terms (28), which constituted exploratory analyses. Second, to account for potential biases introduced by missing data, we employed multiple imputation using the mi package in R (29, 30). The imputation model incorporated all baseline covariates, the exposure variable (preoperative PWR), as well as the outcome (30-day mortality) and time-to-event variables. We generated five complete datasets by running 4 independent chains for 30 iterations. The convergence of the imputation algorithm was confirmed using the Gelman-Rubin diagnostic. The non-linear threshold effect analyses were subsequently repeated across these imputed datasets to verify the stability of our results. Third, we evaluated the influence of extreme PWR values by repeating the main analyses after re-incorporating the 15 participants who had been initially excluded due to outlier status. Fourth, to address potential selection bias arising from the exclusion of patients lacking preoperative PLT and WBC values within the 2-day window, we compared the baseline characteristics and 30-day mortality between the included and excluded cohorts. Furthermore, we repeated the primary Cox proportional hazards regression analysis and the threshold effect analysis in an expanded cohort that included all patients with available preoperative PWR values (*n* = 18,031). In addition, given that the PH assumption for the Cox models was not met, we conducted multivariable logistic regression analyses for 30-day mortality as a supplementary sensitivity analysis.

Data analyses were conducted using EmpowerStats (X&Y Solutions, Inc., Boston, MA) in conjunction with R version 4.2.0. All statistical tests were two-tailed, with a *p* value of less than 0.05 deemed statistically significant.

## Results

### Baseline characteristics

A total of 9,434 adult patients undergoing intracranial tumor craniotomy were included in this retrospective study. Their baseline demographic and clinical characteristics, stratified by preoperative PWR quartiles, are presented in Table 1. The cohort included 81.207% of patients aged 41–80 years and 50.731% female patients. Significant differences across PWR quartiles were observed for many variables, including age range, sex, and comorbidities such as hypertension, diabetes, and disseminated cancer. Similarly, laboratory parameters including BUN, creatinine, sodium, and HCT also demonstrated significant differences across these groups. These findings collectively highlight the significant heterogeneity of baseline characteristics across different preoperative PWR levels.

**Table 1:** Baseline characteristics of study participants by PWR.

Variables	Overall	PWR quartile				
		Q1 (0.659–18.031)	Q2 (18.050–24.674)	Q3 (24.678–33.455)	Q4 (33.457–100.000)	*p* value
*N* (cases)	9,434	2,359	2,358	2,358	2,359	
Age range, *n* (%)						<0.001
18–40 years	1,445 (15.317)	293 (12.421)	356 (15.098)	370 (15.691)	426 (18.058)	
41–60 years	3,898 (41.319)	894 (37.897)	945 (40.076)	983 (41.688)	1,076 (45.613)	
61–80 years	3,763 (39.888)	1,070 (45.358)	974 (41.306)	919 (38.974)	800 (33.913)	
≥ 81 years	328 (3.477)	102 (4.324)	83 (3.520)	86 (3.647)	57 (2.416)	
Sex, *n* (%)						<0.001
Female	4,786 (50.731)	890 (37.728)	1,160 (49.194)	1,270 (53.859)	1,466 (62.145)	
Male	4,648 (49.269)	1,469 (62.272)	1,198 (50.806)	1,088 (46.141)	893 (37.855)	
Race, *n* (%)						0.006
White	6,680 (70.808)	1,685 (71.429)	1,691 (71.713)	1,679 (71.204)	1,625 (68.885)	
Black	695 (7.367)	162 (6.867)	168 (7.125)	148 (6.277)	217 (9.199)	
Other	2059 (21.825)	512 (21.704)	499 (21.162)	531 (22.519)	517 (21.916)	
Severe COPD, *n* (%)						0.271
No	8,965 (95.029)	2,228 (94.447)	2,235 (94.784)	2,254 (95.589)	2,248 (95.295)	
Yes	469 (4.971)	131 (5.553)	123 (5.216)	104 (4.411)	111 (4.705)	
Hypertension, *n* (%)						<0.001
No	5,780 (61.268)	1,296 (54.939)	1,431 (60.687)	1,461 (61.959)	1,592 (67.486)	
Yes	3,654 (38.732)	1,063 (45.061)	927 (39.313)	897 (38.041)	767 (32.514)	
Diabetes, *n* (%)						<0.001
No	8,281 (87.778)	2029 (86.011)	2069 (87.744)	2049 (86.896)	2,134 (90.462)	
Yes	1,153 (12.222)	330 (13.989)	289 (12.256)	309 (13.104)	225 (9.538)	
Systemic infection, *n* (%)						<0.001
No	8,811 (93.396)	2067 (87.622)	2,191 (92.918)	2,258 (95.759)	2,295 (97.287)	
Yes	623 (6.604)	292 (12.378)	167 (7.082)	100 (4.241)	64 (2.713)	
Open wound infection, *n* (%)						0.527
No	9,331 (98.908)	2,331 (98.813)	2,333 (98.940)	2,338 (99.152)	2,329 (98.728)	
Yes	103 (1.092)	28 (1.187)	25 (1.060)	20 (0.848)	30 (1.272)	
Disseminated cancer, *n* (%)						<0.001
No	6,883 (72.960)	1,654 (70.114)	1,677 (71.120)	1774 (75.233)	1778 (75.371)	
Yes	2,551 (27.040)	705 (29.886)	681 (28.880)	584 (24.767)	581 (24.629)	
Bleeding disorders, *n* (%)						<0.001
No	9,180 (97.308)	2,263 (95.930)	2,305 (97.752)	2,296 (97.371)	2,316 (98.177)	
Yes	254 (2.692)	96 (4.070)	53 (2.248)	62 (2.629)	43 (1.823)	
Steroid use, n (%)						<0.001
No	8,147 (86.358)	1880 (79.695)	2039 (86.472)	2073 (87.913)	2,155 (91.352)	
Yes	1,287 (13.642)	479 (20.305)	319 (13.528)	285 (12.087)	204 (8.648)	
Emergency case, *n* (%)						0.134
No	8,462 (89.697)	2091 (88.639)	2,108 (89.398)	2,135 (90.543)	2,128 (90.208)	
Yes	972 (10.303)	268 (11.361)	250 (10.602)	223 (9.457)	231 (9.792)	
Recent weight loss, *n* (%)						0.600
No	9,159 (97.085)	2,298 (97.414)	2,285 (96.904)	2,292 (97.201)	2,284 (96.821)	
Yes	275 (2.915)	61 (2.586)	73 (3.096)	66 (2.799)	75 (3.179)	
Functional health status, *n* (%)						0.242
Independent	8,885 (94.602)	2,206 (93.832)	2,233 (95.143)	2,223 (94.717)	2,223 (94.717)	
Dependent	507 (5.398)	145 (6.168)	114 (4.857)	124 (5.283)	124 (5.283)	
Ventilator, *n* (%)						<0.001
No	9,230 (97.838)	2,263 (95.930)	2,305 (97.752)	2,330 (98.813)	2,332 (98.855)	
Yes	204 (2.162)	96 (4.070)	53 (2.248)	28 (1.187)	27 (1.145)	
ASA classification, *n* (%)						<0.001
Class I-II	2013 (21.589)	372 (15.918)	457 (19.597)	538 (23.110)	646 (27.761)	
Class III-V	7,311 (78.411)	1965 (84.082)	1875 (80.403)	1790 (76.890)	1,681 (72.239)	
BUN, mg/dL, median (Q1-Q3)	16.807 (12.325–22.000)	20.000 (15.000–25.000)	18.000 (13.165–23.000)	16.000 (12.000–20.000)	14.000 (11.000–18.000)	<0.001
Creatinine, mg/dL, median (Q1–Q3)	0.800 (0.679–0.939)	0.810 (0.700–0.996)	0.800 (0.680–0.960)	0.800 (0.670–0.920)	0.770 (0.650–0.900)	<0.001
Sodium, mmol/L, mean ± SD	138.327 ± 3.340	137.610 ± 3.626	138.283 ± 3.280	138.587 ± 3.179	138.836 ± 3.126	<0.001
HCT, %, mean ± SD	39.652 ± 5.070	40.268 ± 5.388	39.923 ± 4.942	39.704 ± 4.892	38.714 ± 4.910	<0.001
PWR	27.044 ± 12.564	13.877 ± 3.109	21.302 ± 1.932	28.851 ± 2.522	44.145 ± 10.464	<0.001

In the study involving 9,434 participants, the proportion of missing data for the variables under examination was 42 (0.445%) for functional health status, 110 (1.166%) for ASA classification, 432 (4.579%) for BUN, 129 (1.367%) for Creatinine, and 145 (1.537%) for sodium. ASA, American Society of Anesthesiologists; BUN, blood urea nitrogen; COPD, chronic obstructive pulmonary disease; HCT, hematocrit; PWR, platelet to white blood cell ratio; SD, standard deviation.

### 30-day postoperative mortality

The overall 30-day mortality rate for the cohort was 3.148% (297/9,434). Significant differences in 30-day mortality were observed across PWR quartiles (*p* < 0.001), with the highest mortality rate in Q1 (5.511%). The lowest mortality rate was observed in Q3 (2.120%, 50/2,358) and Q4 (2.120%, 50/2,359).

### Univariate analysis of 30-day postoperative mortality

As detailed in the univariate Cox regression analysis (Supplementary Table S2), multiple factors were significantly associated with 30-day postoperative mortality. These encompassed demographic characteristics (e.g., older age groups and male sex), a range of comorbidities (including severe COPD, hypertension, diabetes, and disseminated cancer), and clinical severity factors (such as dependent functional health status, emergency case, and ASA classification III-V) (all *p* < 0.05). Furthermore, baseline laboratory parameters, including BUN, creatinine, sodium, and HCT, were also significantly associated with the outcome. Notably, the preliminary unadjusted analysis confirmed that preoperative PWR was significantly associated with 30-day mortality.

### Association between preoperative PWR and 30-day postoperative mortality

The association between preoperative PWR and postoperative 30-day mortality was assessed using Cox proportional hazards models, with results presented in Table 2. In the crude model, a higher preoperative PWR was significantly associated with a decreased risk of 30-day mortality (HR = 0.965, 95% CI: 0.954–0.976, *p* < 0.0001). After adjusting for demographic variables (Model 1), this inverse association remained significant (HR = 0.972, 95% CI: 0.960–0.983, *p* < 0.0001). Further adjustment for additional clinical covariates (Model 2), showed that preoperative PWR continued to be significantly inversely associated with 30-day mortality (HR = 0.978, 95% CI: 0.967–0.990, *p* = 0.0002). When PWR was categorized into quartiles, patients in higher quartiles consistently exhibited a lower risk of 30-day mortality compared to those in the lowest quartile (Q1). Specifically, in the multivariable adjusted Model 2, the HRs for Q2, Q3, and Q4 were 0.586 (95% CI: 0.431–0.796, *p* = 0.0006), 0.515 (95% CI: 0.366–0.724, *p* = 0.0001), and 0.517 (95% CI: 0.360–0.741, *p* = 0.0003), respectively. Moreover, a notable trend of decreasing HRs was observed across the quartiles as the PWR increased (*p* for trend = 0.002).

**Table 2:** The association between preoperative PWR and 30-day postoperative mortality in adult tumor craniotomy.

Exposure	Crude model		Model 1		Model 2	
	HR (95% CI)	*p* value	HR (95% CI)	*p* value	HR (95% CI)	*p* value
PWR	0.965 (0.954, 0.976)	<0.0001	0.972 (0.960, 0.983)	<0.0001	0.978 (0.967, 0.990)	0.0002
PWR quartile						
Q1 (0.659–18.031)	1.00 (Reference)		1.00 (Reference)		1.00 (Reference)	
Q2 (18.050–24.674)	0.507 (0.378, 0.681)	<0.0001	0.544 (0.405, 0.732)	<0.0001	0.586 (0.431, 0.796)	0.0006
Q3 (24.678–33.455)	0.378 (0.273, 0.523)	<0.0001	0.414 (0.298, 0.575)	<0.0001	0.515 (0.366, 0.724)	0.0001
Q4 (33.457–100.000)	0.377 (0.272, 0.523)	<0.0001	0.452 (0.324, 0.631)	<0.0001	0.517 (0.360, 0.741)	0.0003
*P* for trend	<0.0001		<0.0001		0.0002	

Model 1: adjusted for age range, sex and race.

Model 2: adjusted for age range, sex, race, severe chronic obstructive pulmonary disease, hypertension, diabetes, systemic infection, open wound infection, disseminated cancer, bleeding disorders, steroid use, emergency case, recent weight loss, functional health status, ventilator, American Society of Anesthesiologists classification, blood urea nitrogen, creatinine, sodium and hematocrit.

CI, confidence interval; HR, hazard ratio; PWR, platelet to white blood cell ratio.

**Figure 2:**
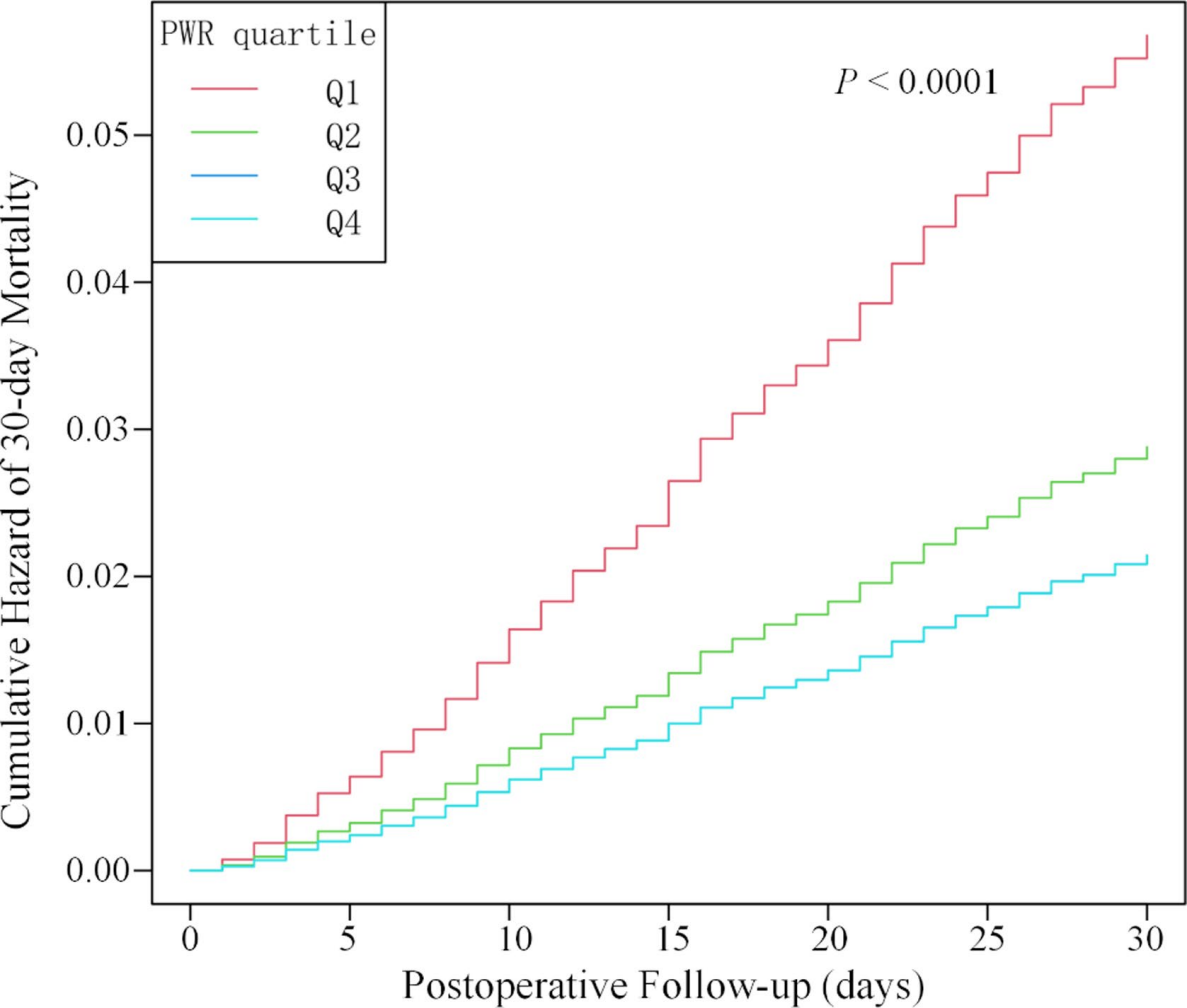
The cumulative hazard curve of 30-day postoperative mortality stratified by preoperative PWR quartile. Kaplan–Meier curves illustrate the cumulative hazard of 30-day mortality for each PWR quartile. A significant difference was observed among the groups (Log-rank *p* < 0.0001). The red line represents PWR Q1, the green line represents PWR Q2, and the blue and light blue lines represent PWR Q3 and Q4, respectively, which largely overlap, indicating similar cumulative hazard profiles for these two quartiles. PWR, platelet to white blood cell ratio.

As displayed in Figure 2, Kaplan–Meier curves showed that patients in higher PWR quartiles (Q2, Q3, and Q4) had a lower cumulative hazard of 30-day mortality compared to those in the lowest quartile (Q1) (*p* < 0.0001).

### Non-linear relationship between preoperative PWR and 30-day postoperative mortality

**Figure 3:**
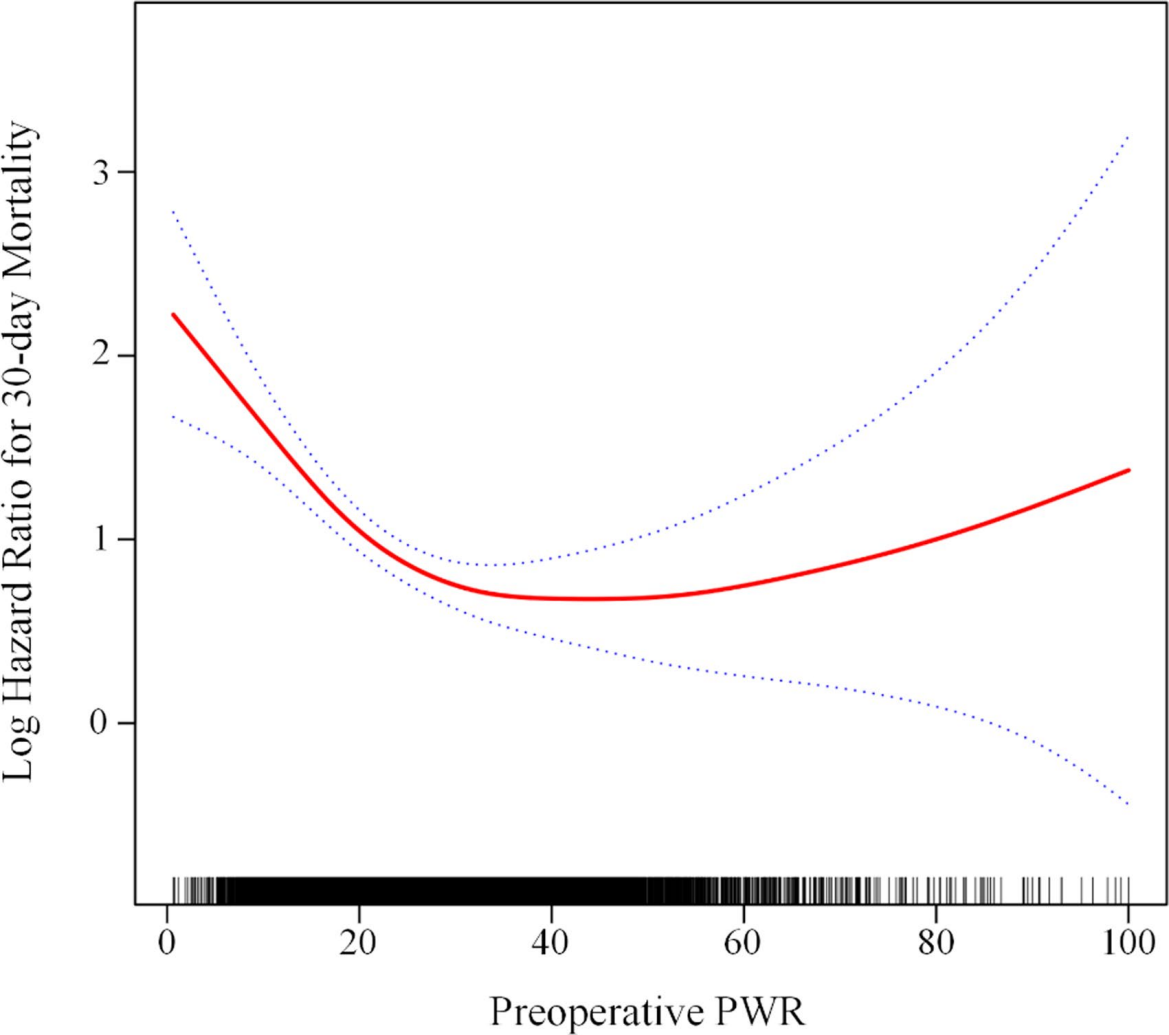
The non-linear relationship between preoperative PWR and 30-day postoperative mortality. The solid red line denotes the smoothed curve fit between the variables, while the blue bands illustrate the 95% confidence interval associated with this fit. Adjusted for age range, sex, race, severe chronic obstructive pulmonary disease, hypertension, diabetes, systemic infection, open wound infection, disseminated cancer, bleeding disorders, steroid use, emergency case, recent weight loss, functional health status, ventilator, American Society of Anesthesiologists classification, blood urea nitrogen, creatinine, sodium and hematocrit. PWR, platelet to white blood cell ratio.

As illustrated in Figure 3, preoperative PWR and 30-day postoperative mortality exhibited a non-linear relationship. Utilizing a two-piecewise Cox regression model, we determined the inflection point for preoperative PWR was 20.111 (Table 3), supported by a significant LRT (*p* < 0.001). Below this value, each unit increase in preoperative PWR was associated with an approximate 8% decrease in the risk of 30-day mortality (HR = 0.920, 95% CI: 0.892–0.949, *p* < 0.0001). Above the value, the relationship tended to be saturated (HR = 0.997, 95% CI: 0.984–1.011, *p* = 0.6985).

**Table 3:** Threshold effect analysis of preoperative PWR and 30-day postoperative mortality.

Models	HR (95% CI)	*p* value
Model I		
One line effect	0.978 (0.967, 0.990)	0.0002
Model II		
Turning point (K)	20.111	
PWR < K	0.920 (0.892, 0.949)	<0.0001
PWR ≥ K	0.997 (0.984, 1.011)	0.6985
*p* value for LRT test^*^	<0.001	

Model I, linear analysis; Model II, non-linear analysis.

Adjusted for age range, sex, race, severe chronic obstructive pulmonary disease, hypertension, diabetes, systemic infection, open wound infection, disseminated cancer, bleeding disorders, steroid use, emergency case, recent weight loss, functional health status, ventilator, American Society of Anesthesiologists classification, blood urea nitrogen, creatinine, sodium and hematocrit.

^*^p < 0.05 suggests a statistically significant difference between Model II and Model I.

CI, confidence interval; HR, hazard ratio; LRT, log-likelihood ratio test; PWR, platelet to white blood cell ratio.

### Sensitivity analysis

To assess the robustness of our primary findings, we performed several sensitivity analyses. First, we addressed potential population heterogeneity by conducting exploratory stratified analyses for the association between preoperative PWR and 30-day mortality across various subgroups, as detailed in Table 4. The inverse association between preoperative PWR and 30-day mortality generally remained consistent across most subgroups, including age range, sex, race, and various comorbidities such as hypertension, diabetes, and systemic infection. In these exploratory subgroup analyses, while acknowledging the increased likelihood of chance findings from multiple interaction tests, most interactions were not statistically significant, suggesting the general robustness of our main findings.

**Table 4:** Sensitivity analysis: stratified associations between preoperative PWR and 30-day postoperative mortality in adult tumor craniotomy.

Stratified variable		Events (%)	HR (95% CI)	*p* value	*p* for interaction^*^
Age range	18–40 years	15 (1.038)	0.986 (0.940, 1.035)	0.5740	0.5566
	41–60 years	98 (2.514)	0.979 (0.960, 1.000)	0.0450	
	61–80 years	151 (4.013)	0.981 (0.966, 0.997)	0.0193	
	≥ 81 years	33 (10.061)	0.963 (0.923, 1.005)	0.0872	
Sex	Female	123 (2.570)	0.976 (0.959, 0.994)	0.0076	0.5209
	Male	174 (3.744)	0.979 (0.964, 0.994)	0.0062	
Race	White	204 (3.054)	0.978 (0.965, 0.992)	0.0018	0.6158
	Black	16 (2.302)	0.990 (0.951, 1.031)	0.6419	
	Other	77 (3.740)	0.971 (0.947, 0.996)	0.0216	
Severe COPD	No	272 (3.034)	0.976 (0.964, 0.988)	<0.0001	0.1787
	Yes	25 (5.330)	1.010 (0.976, 1.045)	0.5872	
Hypertension	No	123 (2.128)	0.984 (0.968, 1.000)	0.0505	0.6360
	Yes	174 (4.762)	0.972 (0.957, 0.988)	0.0007	
Diabetes	No	229 (2.765)	0.976 (0.963, 0.989)	0.0004	0.5305
	Yes	68 (5.898)	0.984 (0.961, 1.008)	0.1916	
Systemic infection	No	262 (2.974)	0.976 (0.964, 0.988)	0.0001	0.2362
	Yes	35 (5.618)	0.985 (0.960, 1.010)	0.2426	
Open wound infection	No	283 (3.033)	0.977 (0.965, 0.989)	0.0001	0.5223
	Yes	14 (13.592)	1.002 (0.952, 1.055)	0.9256	
Disseminated cancer	No	149 (2.165)	0.967 (0.950, 0.985)	0.0003	0.0940
	Yes	148 (5.802)	0.988 (0.974, 1.003)	0.1097	
Bleeding disorders	No	281 (3.061)	0.980 (0.968, 0.991)	0.0007	0.3887
	Yes	16 (6.299)	0.946 (0.878, 1.021)	0.1535	
Steroid use	No	217 (2.664)	0.973 (0.960, 0.987)	0.0002	0.2255
	Yes	80 (6.216)	0.990 (0.970, 1.010)	0.3116	
Emergency case	No	235 (2.777)	0.977 (0.964, 0.990)	0.0006	0.3642
	Yes	62 (6.379)	0.983 (0.961, 1.006)	0.1439	
Recent weight loss	No	266 (2.904)	0.972 (0.959, 0.985)	<0.0001	0.0371
	Yes	31 (11.273)	1.003 (0.978, 1.030)	0.8074	
Functional health status	Independent	241 (2.712)	0.978 (0.966, 0.991)	0.0007	0.9243
	Dependent	50 (9.862)	0.982 (0.955, 1.009)	0.1880	
Ventilator	No	276 (2.990)	0.978 (0.966, 0.990)	0.0003	0.5382
	Yes	21 (10.294)	0.990 (0.946, 1.036)	0.6563	
ASA classification	Class I-II	12 (0.596)	1.008 (0.961, 1.058)	0.7332	0.1949
	Class III-V	285 (3.898)	0.977 (0.965, 0.989)	0.0001	
BUN	Low	47 (1.682)	0.996 (0.973, 1.020)	0.7617	0.4145
	Middle	87 (2.751)	0.974 (0.952, 0.995)	0.0179	
	High	158 (5.187)	0.974 (0.957, 0.990)	0.0020	
Creatinine	Low	71 (2.757)	0.992 (0.971, 1.012)	0.4242	0.2139
	Middle	109 (3.059)	0.987 (0.968, 1.005)	0.1627	
	High	117 (3.694)	0.960 (0.940, 0.981)	0.0002	
Sodium	Low	119 (5.000)	0.973 (0.955, 0.992)	0.0047	0.3356
	Middle	86 (2.418)	0.971 (0.948, 0.994)	0.0130	
	High	92 (2.745)	0.991 (0.971, 1.011)	0.3514	
HCT	Low	141 (4.568)	0.994 (0.981, 1.008)	0.4280	0.0188
	Middle	80 (2.597)	0.952 (0.926, 0.979)	0.0005	
	High	76 (2.326)	0.964 (0.936, 0.993)	0.0168	

The multivariate model adjusted for age range, sex, race, severe COPD, hypertension, diabetes, systemic infection, open wound infection, disseminated cancer, bleeding disorders, steroid use, emergency case, recent weight loss, functional health status, ventilator, ASA classification, BUN, creatinine, sodium and HCT, except for the variable that is stratified. BUN, creatinine, sodium and HCT were categorized into tertiles.

^*^The interaction tests presented in this table are exploratory.

ASA, American Society of Anesthesiologists; BUN, blood urea nitrogen; COPD, chronic obstructive pulmonary disease; CI, confidence interval; HCT, hematocrit; HR, hazard ratio; PWR, platelet to white blood cell ratio.

Second, we employed multiple imputation to address potential bias from missing data. The relationship between preoperative PWR and postoperative 30-day mortality persisted as statistically significant across all five imputed datasets, exhibiting consistent effect sizes (Supplementary Table S3). The pooled HR of 0.979 (95% CI: 0.968–0.990) was highly consistent with that from the pre-imputation analysis (HR = 0.978, 95% CI: 0.967–0.990). Moreover, the threshold effect analysis remained statistically significant in every imputed dataset, with the turning point of preoperative PWR consistently ranging from 20.135 to 20.156 (all *p* values for LRT were < 0.05) (Supplementary Table S4).

Third, to assess the impact of excluding PWR outliers, we performed additional analyses by including the 15 participants initially removed due to extreme PWR values. In this analytical cohort (n = 9,449), the association between preoperative PWR and 30-day mortality, both as a continuous variable and when categorized by quartiles, remained statistically significant and consistent with our primary findings (Supplementary Table S5). Furthermore, the threshold effect analysis also showed robust results, with the turning point and corresponding HRs closely mirroring those from the main analysis (Supplementary Table S6).

Fourth, we compared the baseline characteristics between the 9,434 included patients and the 9,208 excluded patients (Supplementary Table S7). The analysis revealed significant differences between the two groups. Notably, the included analytic cohort exhibited a higher 30-day postoperative mortality rate (3.148% vs. 1.748%, *p* < 0.0001) and a generally higher clinical burden, including greater proportions of ASA classification III-V, diabetes, and disseminated cancer. Nevertheless, we repeated the main analyses in all patients with available preoperative PWR values (*n* = 18,031). In this expanded cohort, the inverse association between preoperative PWR and 30-day postoperative mortality remained statistically significant in the multivariable adjusted model (HR = 0.974, 95% CI: 0.965–0.983, *p* < 0.0001) (Supplementary Table S8), and the threshold effect analysis also confirmed the non-linear relationship (*p* for LRT < 0.001, Supplementary Table S9), supporting the robustness of our primary findings.

Finally, considering the violation of the PH assumption, we employed multivariable logistic regression models to re-evaluate the association. The results remained consistent with our primary findings. Preoperative PWR continued to exhibit a significant inverse association with 30-day mortality (Model 2: odds ratio = 0.978, 95% CI: 0.966–0.990, *p* = 0.0003) (Supplementary Table S10). The non-linear relationship was also preserved (*p* for LRT < 0.001, Supplementary Table S11).

## Discussion

In this retrospective cohort study utilizing the ACS NSQIP database (2012–2015), we investigated the association between preoperative PWR and 30-day postoperative mortality in adults undergoing intracranial tumor craniotomy. Our findings demonstrate a significant, non-linear inverse relationship between preoperative PWR levels and the risk of 30-day mortality. Our study adds to the current literature by highlighting this non-linear association in a large cohort of U. S. adults undergoing this procedure. These findings warrant confirmation in future research.

Although no previous studies have specifically investigated the association between preoperative PWR and short-term mortality in patients undergoing intracranial tumor craniotomy, our findings align closely with recent evidence derived from other high-risk clinical populations. Consistent with our results, which demonstrated a significant inverse association and a progressively decreased mortality risk across higher PWR quartiles, prior research has similarly linked elevated PWR to a reduced risk of adverse short-term outcomes (10, 13). For instance, a recent large-scale study of critically ill patients with atherosclerotic cardiovascular disease reported a significantly lower risk of 30-day mortality in the highest PWR quartile (10). Similarly, a cohort study of patients with acute decompensated heart failure observed a negative association between PWR and 30-day all-cause mortality (13). Furthermore, the non-linear dose–response pattern we observed is strongly corroborated by the acute decompensated heart failure study, which also demonstrated a similar L-shaped association characterized by a threshold effect. Below this threshold, the mortality risk decreased substantially as PWR increased. This consistency suggests that the non-linear relationship between PWR and short-term mortality may reflect shared pathophysiological underpinnings, further reinforcing the reliability of the threshold effect observed in our neurosurgical cohort.

Building upon these putative shared pathophysiological underpinnings, the observed association between PWR and mortality extends beyond cardiovascular populations to neurosurgical settings. For instance, in patients with traumatic acute subdural hematoma, a decreased PWR has been independently associated with an increased incidence of acute brain swelling and higher mortality rates (31). As a ratio mathematically integrating PLT count in the numerator and WBC count in the denominator, PWR inherently reflects concurrent alterations in hemostatic function and inflammatory activity within a single metric (11, 12). Therefore, PWR serves as a composite index that can directly integrate these concurrent hematological alterations. By capturing the delicate balance between the inflammatory and coagulation systems (10), this integrated index provides a more comprehensive reflection of the patient’s preoperative physiological vulnerability, thereby providing a biologically plausible explanation for the inverse association with short-term mortality observed in our cohort.

While the observational design of our study constrains our capacity to establish definitive causal inferences, the existing literature offers several biologically plausible mechanisms that may elucidate the observed associations. First, a lower PWR can be driven by an elevated leukocyte count, which typically reflects a pronounced systemic inflammatory state. In neurosurgical contexts, an exaggerated preoperative inflammatory burden could signify a heightened susceptibility to severe postoperative complications, such as SIRS or sepsis, which are established risk factors for short-term mortality (19). Additionally, pronounced systemic inflammation might also serve as a surrogate index for more advanced intracranial tumor grades or aggressive disease biology, which inherently carry a higher baseline surgical risk (32, 33). Second, the numerator of the PWR highlights the critical role of hemostatic reserve. A decreased PWR implies a relative reduction in circulating platelets. Adequate platelet count and function are not only vital for maintaining endothelial integrity and preventing hemorrhagic complications (10, 34), but they also actively modulate local immune signaling and facilitate tissue repair (35, 36). When platelet reserves are depleted, patients may become more vulnerable to microvascular leakage and impaired cerebral tissue recovery following surgical trauma. Third, the interplay between leukocytes and platelets, may directly exacerbate postoperative injury. Evidence suggests that activated platelets can adhere to leukocytes, forming leukocyte-platelet aggregates (37, 38). This process not only consumes circulating platelets but also heavily recruits inflammatory cells into the vasculature. In the context of intracranial surgery, this dysregulated crosstalk may promote microvascular plugging and amplify secondary brain injury in the vulnerable brain tissue (39, 40). Consequently, a severely depressed PWR may collectively mirror an overactive systemic inflammatory response coupled with a depleted hemostatic reserve, providing a biologically plausible explanation for the increased risk of short-term mortality observed in our study.

Several limitations should be acknowledged when interpreting the results of this study. First, due to the retrospective observational design, we cannot infer definitive causality between preoperative PWR levels and 30-day postoperative mortality. Future prospective cohorts are required to confirm these findings and formally evaluate causal links. Second, while our multivariable models adjusted for a wide array of clinical covariates, the inherent nature of a secondary analysis utilizing a clinical registry means that residual confounding remains a possibility. Specifically, important tumor-related and treatment-related variables were unavailable in the dataset (19), including tumor histopathology, grade, location, volume, extent of resection, preoperative performance status, specific medication exposure (such as antiplatelet or anticoagulant use), steroid dosage, and perioperative transfusion. The absence of these variables inevitably limits our ability to address the underlying heterogeneity of our cohort. As different tumor types inherently exert distinct biological and surgical effects on patient outcomes (41), the lack of detailed subgroup analyses according to tumor histopathology limits a more nuanced interpretation of our findings. Consequently, without these critical variables, it is difficult to definitively determine whether PWR acts as an independent factor or simply serves as a surrogate for overall illness severity. Third, beyond the aforementioned clinical and pathological variables, our study lacks highly granular neuro-radiological features and anatomical parameters. Crucially, factors such as the degree of peritumoral edema, perioperative bleeding tendency, tumor vascularity, proximity to major vascular structures, and overall surgical complexity were not captured in the registry. These specific anatomical and surgical challenges may substantially influence clinical outcomes and surgical morbidity (42, 43). Consequently, the absence of these variables precludes comprehensive risk adjustment, making it difficult to determine whether the observed association between PWR and mortality is independent of these inherent surgical complexities. Future prospective studies incorporating comprehensive radiological assessments and standardized surgical complexity scores are essential to address these unmeasured risks. Fourth, missing values for certain covariates and the exclusion of patients lacking recent preoperative exposure data (within 2 days prior to surgery) introduced information and selection bias. Our comparison revealed that the included patients were generally sicker than the excluded ones. However, we mitigated this risk by applying multiple imputation for covariates and, more importantly, by performing a sensitivity analysis in all patients with available PWR data. Reassuringly, the non-linear inverse association between PWR and 30-day mortality retained its statistical significance across all imputed datasets and the expanded cohort, confirming the robustness of our main findings. Fifth, the current study was designed exclusively as an observational association analysis to explore the relationship between preoperative PWR and 30-day postoperative mortality. We did not evaluate the predictive performance of PWR (such as discrimination, calibration, or decision-curve metrics), nor did we compare it against established multi-parameter clinical prediction models. Consequently, the current findings do not support the clinical utility of PWR as an independent risk stratification tool. The clinical interpretation of these results should be cautious, and whether PWR can provide actionable predictive value requires further validation in rigorously designed prognostic studies. Furthermore, the subgroup analyses presented in this study were exploratory in nature. Several subgroups contained limited numbers of outcome events, and multiple interaction tests increase the likelihood of chance findings. Therefore, the subgroup results should not be interpreted as definitive evidence of effect modification. Finally, because the dataset is predominantly derived from participating hospitals within the United States, the generalizability of our findings to patients in other geographic regions or varying healthcare settings may be restricted.

Despite the observational nature of this study, our findings carry potential clinical implications that merit further investigation. While the current study primarily establishes a novel non-linear association in a neuro-oncological cohort, future prospective research could explore focus on the potential clinical applicability of this parameter. Specifically, it is crucial to investigate how integrating preoperative PWR might influence patient management, guide perioperative decision-making, and improve prognostic stratification in daily practice. For instance, evaluating whether patients with a PWR below the observed threshold might benefit from closer perioperative monitoring or tailored risk assessments represents a highly clinically relevant direction for future investigations.

## Conclusion

This study, which analyzed a cohort of 9,434 U. S. adults who underwent craniotomy for intracranial tumors using data from the ACS NSQIP, demonstrated a significant non-linear inverse association between preoperative PWR levels and the risk of 30-day postoperative mortality. Additional research is required to confirm these observational findings.

## Data Availability

Publicly available datasets were analyzed in this study. This data can be found at: https://doi.org/10.1371/journal.pone.0235273.
