# Supplementary material for: The non-linear association between preoperative platelet to white blood cell ratio and 30-day postoperative mortality in adult tumor craniotomy: a retrospective cohort study
Source: Front Neurol. 2026 Jul 20;17:1852566. doi: 10.3389/fneur.2026.1852566 (PMC13429503; doi:10.3389/fneur.2026.1852566)
Supplement: Supplementary file 1 [file Table_1.docx]

Supplementary Material

# Supplementary Tables

TABLE S1 CPT codes and corresponding surgical procedures for tumor craniotomy.

| **CPT codes** | **Surgical procedures** |
| --- | --- |
| 61510 | Supratentorial craniotomy for tumor |
| 61512 | Supratentorial craniotomy for meningioma |
| 61518 | Infratentorial craniotomy for tumor: others |
| 61519 | Infratentorial craniotomy for meningioma |
| 61520 | Infratentorial craniotomy for tumor: cerebellopontine angle |
| 61521 | Infratentorial craniotomy for tumor: midline |
| 61526 | Trans labyrinthine approach for cerebellopontine angle tumor |
| 61545 | Craniotomy for craniopharyngioma |
| 61546 | Craniotomy for pituitary macroadenoma |
| 61575 | Transoral approach to skull base, brain stem, or upper spinal cord for biopsy, decompression,or excision of lesion |

CPT, Current Procedural Terminology.

**TABLE S2 The univariate analysis of 30-day postoperative mortality.**

| **Variables** | **Statistics** | **HR (95% CI)** | P value |
| --- | --- | --- | --- |
| Age range |  |  |  |
| 18-40 years | 1445 (15.317%) | 1.00 (Reference) |  |
| 41-60 years | 3898 (41.319%) | 2.438 (1.416, 4.197) | 0.0013 |
| 61-80 years | 3763 (39.888%) | 3.922 (2.307, 6.667) | <0.0001 |
| ≥ 81 years | 328 (3.477%) | 10.197 (5.538, 18.773) | <0.0001 |
| Sex |  |  |  |
| Female | 4786 (50.731%) | 1.00 (Reference) |  |
| Male | 4648 (49.269%) | 1.462 (1.160, 1.841) | 0.0013 |
| Race |  |  |  |
| White | 6680 (70.808%) | 1.00 (Reference) |  |
| Black | 695 (7.367%) | 0.752 (0.452, 1.250) | 0.2718 |
| Other | 2059 (21.825%) | 1.230 (0.946, 1.598) | 0.1224 |
| Severe COPD |  |  |  |
| No | 8965 (95.029%) | 1.00 (Reference) |  |
| Yes | 469 (4.971%) | 1.771 (1.176, 2.668) | 0.0062 |
| Hypertension |  |  |  |
| No | 5780 (61.268%) | 1.00 (Reference) |  |
| Yes | 3654 (38.732%) | 2.264 (1.797, 2.851) | <0.0001 |
| Diabetes |  |  |  |
| No | 8281 (87.778%) | 1.00 (Reference) |  |
| Yes | 1153 (12.222%) | 2.170 (1.655, 2.844) | <0.0001 |
| Systemic infection |  |  |  |
| No | 8811 (93.396%) | 1.00 (Reference) |  |
| Yes | 623 (6.604%) | 1.923 (1.351, 2.736) | 0.0003 |
| Open wound infection |  |  |  |
| No | 9331 (98.908%) | 1.00 (Reference) |  |
| Yes | 103 (1.092%) | 4.711 (2.754, 8.057) | <0.0001 |
| Disseminated cancer |  |  |  |
| No | 6883 (72.960%) | 1.00 (Reference) |  |
| Yes | 2551 (27.040%) | 2.723 (2.169, 3.418) | <0.0001 |
| Bleeding disorders |  |  |  |
| No | 9180 (97.308%) | 1.00 (Reference) |  |
| Yes | 254 (2.692%) | 2.106 (1.273, 3.485) | 0.0038 |
| Steroid use |  |  |  |
| No | 8147 (86.358%) | 1.00 (Reference) |  |
| Yes | 1287 (13.642%) | 2.372 (1.835, 3.065) | <0.0001 |
| Emergency case |  |  |  |
| No | 8462 (89.697%) | 1.00 (Reference) |  |
| Yes | 972 (10.303%) | 2.344 (1.772, 3.101) | <0.0001 |
| Recent weight loss |  |  |  |
| No | 9159 (97.085%) | 1.00 (Reference) |  |
| Yes | 275 (2.915%) | 4.023 (2.773, 5.835) | <0.0001 |
| Functional health status |  |  |  |
| Independent | 8885 (94.602%) | 1.00 (Reference) |  |
| Dependent | 507 (5.398%) | 3.754 (2.768, 5.091) | <0.0001 |
| Ventilator |  |  |  |
| No | 9230 (97.838%) | 1.00 (Reference) |  |
| Yes | 204 (2.162%) | 3.613 (2.318, 5.631) | <0.0001 |
| ASA classification |  |  |  |
| Class I-II | 2013 (21.589%) | 1.00 (Reference) |  |
| Class III-V | 7311 (78.411%) | 6.654 (3.735, 11.856) | <0.0001 |
| BUN | 18.157 ± 9.085 | 1.028 (1.023, 1.034) | <0.0001 |
| Creatinine | 0.857 ± 0.510 | 1.218 (1.106, 1.341) | 0.0001 |
| Sodium | 138.327 ± 3.340 | 0.927 (0.898, 0.958) | <0.0001 |
| HCT | 39.652 ± 5.070 | 0.918 (0.900, 0.936) | <0.0001 |
| PWR | 27.044 ± 12.564 | 0.965 (0.954, 0.976) | <0.0001 |

Data are expressed as the mean ± standard deviation, or counts with percentages.

ASA, American Society of Anesthesiologists; BUN, blood urea nitrogen; COPD, chronic obstructive pulmonary disease; CI, confidence interval; HCT, hematocrit; HR, hazard ratio; PWR, platelet to white blood cell ratio.

TABLE S3 The association between preoperative PWR and 30-day postoperative mortality across five multiple imputation datasets.

| **Exposure** | **Multiple imputation** | | | | | **Pool estimates  from multiple imputed data** |
| --- | --- | --- | --- | --- | --- | --- |
|  | **Dataset 1** | **Dataset 2** | **Dataset 3** | **Dataset 4** | **Dataset 5** |  |
|  | **HR (95% CI)  *P* value** | **HR (95% CI)  *P* value** | **HR (95% CI)  *P* value** | **HR (95% CI)  *P* value** | **HR (95% CI)  *P* value** | **HR (95% CI)  *P* value** |
| PWR | 0.979 (0.968, 0.990)  0.0002 | 0.979 (0.968, 0.990)  0.0002 | 0.979 (0.968, 0.990)  0.0002 | 0.979 (0.968, 0.990)  0.0002 | 0.979 (0.968, 0.990)  0.0002 | 0.979 (0.968, 0.990)  0.0002 |
| PWR quartile |  |  |  |  |  |  |
| Q1 (0.659-18.031) | 1.00 (Reference) | 1.00 (Reference) | 1.00 (Reference) | 1.00 (Reference) | 1.00 (Reference) | 1.00 (Reference) |
| Q2 (18.050-24.674) | 0.617 (0.456, 0.833)  0.0017 | 0.616 (0.456, 0.832)  0.0016 | 0.616 (0.456, 0.833)  0.0016 | 0.615 (0.455, 0.831)  0.0015 | 0.617 (0.456, 0.833)  0.0017 | 0.616 (0.456, 0.833)  0.0016 |
| Q3 (24.678-33.455) | 0.510 (0.364, 0.714)  <0.0001 | 0.511 (0.364, 0.715)  <0.0001 | 0.511 (0.364, 0.716)  <0.0001 | 0.511 (0.365, 0.716)  0.0001 | 0.510 (0.364, 0.714)  <0.0001 | 0.511 (0.364, 0.715)  <0.0001 |
| Q4 (33.457-100.000) | 0.538 (0.380, 0.763)  0.0005 | 0.536 (0.378, 0.759)  0.0004 | 0.535 (0.378, 0.758)  0.0004 | 0.539 (0.381, 0.764)  0.0005 | 0.537 (0.379, 0.762)  0.0005 | 0.537 (0.379 0.761)  0.0005 |
| *P* for trend | 0.0002 | 0.0002 | 0.0002 | 0.0002 | 0.0002 |  |

Adjusted for age range, sex, race, severe chronic obstructive pulmonary disease, hypertension, diabetes, systemic infection, open wound infection, disseminated cancer, bleeding disorders, steroid use, emergency case, recent weight loss, functional health status, ventilator, American Society of Anesthesiologists classification, blood urea nitrogen, creatinine, sodium and hematocrit.

CI, confidence interval; HR, hazard ratio; PWR, platelet to white blood cell ratio.

TABLE S4 Threshold effect analysis of preoperative PWR and 30-day postoperative mortality across five multiple imputation datasets.

| **Models** | **Multiple imputation  dataset 1** | **Multiple imputation  dataset 2** | **Multiple imputation  dataset 3** | **Multiple imputation  dataset 4** | **Multiple imputation  dataset 5** |
| --- | --- | --- | --- | --- | --- |
|  | **HR (95% CI)  *P* value** | **HR (95% CI)  *P* value** | **HR (95% CI)  *P* value** | **HR (95% CI)  *P* value** | **HR (95% CI)  *P* value** |
| Model I |  |  |  |  |  |
| One line effect | 0.979 (0.968, 0.990)  0.0002 | 0.979 (0.968, 0.990)  0.0002 | 0.979 (0.968, 0.990)  0.0002 | 0.979 (0.968, 0.990)  0.0002 | 0.979 (0.968, 0.990)  0.0002 |
| Model II |  |  |  |  |  |
| Turning point (K) | 20.156 | 20.141 | 20.135 | 20.137 | 20.141 |
| PWR < K | 0.922 (0.894, 0.950)  <0.0001 | 0.921 (0.894, 0.949)  <0.0001 | 0.921 (0.894, 0.949)  <0.0001 | 0.921 (0.893, 0.949)  <0.0001 | 0.921 (0.894, 0.949)  <0.0001 |
| PWR ≥ K | 0.998 (0.985, 1.011)  0.7695 | 0.998 (0.985, 1.011) 0.7672 | 0.998 (0.985, 1.011)  0.7650 | 0.998 (0.985, 1.011)  0.7880 | 0.998 (0.985, 1.011)  0.7648 |
| *P* value for LRT^*^ | <0.001 | <0.001 | <0.001 | <0.001 | <0.001 |

Model I, linear analysis; Model II, non-linear analysis.

Adjusted for age range, sex, race, severe chronic obstructive pulmonary disease, hypertension, diabetes, systemic infection, open wound infection, disseminated cancer, bleeding disorders, steroid use, emergency case, recent weight loss, functional health status, ventilator, American Society of Anesthesiologists classification, blood urea nitrogen, creatinine, sodium and hematocrit.

^*^*P* < 0.05 suggests a statistically significant difference between Model II and Model I.

CI, confidence interval; HR, hazard ratio; LRT, log-likelihood ratio test; PWR, platelet to white blood cell ratio.

TABLE S5 The association between preoperative PWR and 30-day postoperative mortality after including outlier of PWR (n = 9449).

| **Exposure** | **Crude model** | | **Model 1** | | **Model 2** | |
| --- | --- | --- | --- | --- | --- | --- |
|  | **HR (95% CI)** | ***P* value** | **HR (95% CI)** | ***P* value** | **HR (95% CI)** | ***P* value** |
| PWR | 0.965 (0.954, 0.976) | <0.0001 | 0.971 (0.960, 0.983) | <0.0001 | 0.978 (0.967, 0.990) | 0.0002 |
| PWR quartile |  |  |  |  |  |  |
| Q1 (0.659-18.056) | 1.00 (Reference) |  | 1.00 (Reference) |  | 1.00 (Reference) |  |
| Q2 (18.058-24.697) | 0.515 (0.384, 0.691) | <0.0001 | 0.553 (0.412, 0.743) | <0.0001 | 0.594 (0.438, 0.807) | 0.0009 |
| Q3 (24.700-33.478) | 0.370 (0.266, 0.514) | <0.0001 | 0.405 (0.291, 0.564) | <0.0001 | 0.504 (0.357, 0.712) | 0.0001 |
| Q4 (33.488-2810.000) | 0.377 (0.272, 0.523) | <0.0001 | 0.454 (0.325, 0.633) | <0.0001 | 0.517 (0.360, 0.742) | 0.0004 |
| *P* for trend | 0.960 (0.948, 0.973) | <0.0001 | 0.967 (0.954, 0.979) | <0.0001 | 0.973 (0.960, 0.987) | 0.0002 |

Adjusted for age range, sex, race, severe chronic obstructive pulmonary disease, hypertension, diabetes, systemic infection, open wound infection, disseminated cancer, bleeding disorders, steroid use, emergency case, recent weight loss, functional health status, ventilator, American Society of Anesthesiologists classification, blood urea nitrogen, creatinine, sodium and hematocrit.

CI, confidence interval; HR, hazard ratio; PWR, platelet to white blood cell ratio.

TABLE S6 Threshold effect analysis of preoperative PWR and 30-day postoperative mortality after including outlier of PWR (n = 9449).

| **Models** | **HR (95% CI)** | ***P* value** |
| --- | --- | --- |
| Model I |  |  |
| One line effect | 0.978 (0.967, 0.990) | 0.0002 |
| Model II |  |  |
| Turning point (K) | 20.090 |  |
| PWR < K | 0.920 (0.892, 0.949) | <0.0001 |
| PWR ≥ K | 0.997 (0.984, 1.010) | 0.6225 |
| *P* value for LRT test* | <0.001 |  |

Model I, linear analysis; Model II, non-linear analysis.

Adjusted for age range, sex, race, severe chronic obstructive pulmonary disease, hypertension, diabetes, systemic infection, open wound infection, disseminated cancer, bleeding disorders, steroid use, emergency case, recent weight loss, functional health status, ventilator, American Society of Anesthesiologists classification, blood urea nitrogen, creatinine, sodium and hematocrit.

^*^*P* < 0.05 suggests a statistically significant difference between Model II and Model I.

CI, confidence interval; HR, hazard ratio; LRT, log-likelihood ratio test; PWR, platelet to white blood cell ratio.

TABLE S7 Comparison of characteristics between the included and excluded patients.

| **Variables** | **Excluded patients (n = 9208)** | **Included patients (n = 9434)** | ***P* value** |
| --- | --- | --- | --- |
| Age range, n (%) |  |  | <0.001 |
| 18-40 years | 1612 (17.507%) | 1445 (15.317%) |  |
| 41-60 years | 3844 (41.746%) | 3898 (41.319%) |  |
| 61-80 years | 3470 (37.685%) | 3763 (39.888%) |  |
| ≥ 81 years | 282 (3.063%) | 328 (3.477%) |  |
| Sex, n (%) |  |  | <0.001 |
| Female | 5020 (54.518%) | 4786 (50.731%) |  |
| Male | 4188 (45.482%) | 4648 (49.269%) |  |
| Race, n (%) |  |  | <0.001 |
| White | 6610 (71.785%) | 6680 (70.808%) |  |
| Black | 550 (5.973%) | 695 (7.367%) |  |
| Other | 2048 (22.242%) | 2059 (21.825%) |  |
| Severe COPD, n (%) |  |  | 0.001 |
| No | 8842 (96.025%) | 8965 (95.029%) |  |
| Yes | 366 (3.975%) | 469 (4.971%) |  |
| Hypertension, n (%) |  |  | 0.111 |
| No | 5746 (62.402%) | 5780 (61.268%) |  |
| Yes | 3462 (37.598%) | 3654 (38.732%) |  |
| Diabetes, n (%) |  |  | 0.023 |
| No | 8181 (88.847%) | 8281 (87.778%) |  |
| Yes | 1027 (11.153%) | 1153 (12.222%) |  |
| Systemic infection, n (%) |  |  | <0.001 |
| No | 9159 (99.468%) | 8811 (93.396%) |  |
| Yes | 49 (0.532%) | 623 (6.604%) |  |
| Open wound infection, n (%) |  |  | <0.001 |
| No | 9156 (99.435%) | 9331 (98.908%) |  |
| Yes | 52 (0.565%) | 103 (1.092%) |  |
| Disseminated cancer, n (%) |  |  | <0.001 |
| No | 7737 (84.025%) | 6883 (72.960%) |  |
| Yes | 1471 (15.975%) | 2551 (27.040%) |  |
| Bleeding disorders, n (%) |  |  | <0.001 |
| No | 9089 (98.708%) | 9180 (97.308%) |  |
| Yes | 119 (1.292%) | 254 (2.692%) |  |
| Steroid use, n (%) |  |  | <0.001 |
| No | 7696 (83.579%) | 8147 (86.358%) |  |
| Yes | 1512 (16.421%) | 1287 (13.642%) |  |
| Emergency case, n (%) |  |  | <0.001 |
| No | 8982 (97.546%) | 8462 (89.697%) |  |
| Yes | 226 (2.454%) | 972 (10.303%) |  |
| Recent weight loss, n (%) |  |  | <0.001 |
| No | 9078 (98.588%) | 9159 (97.085%) |  |
| Yes | 130 (1.412%) | 275 (2.915%) |  |
| Functional health status, n (%) |  |  | <0.001 |
| Independent | 8901 (97.172%) | 8885 (94.602%) |  |
| Dependent | 259 (2.828%) | 507 (5.398%) |  |
| Ventilator, n (%) |  |  | <0.001 |
| No | 9200 (99.913%) | 9230 (97.838%) |  |
| Yes | 8 (0.087%) | 204 (2.162%) |  |
| ASA classification, n (%) |  |  | <0.001 |
| Class I-II | 3017 (32.965%) | 2013 (21.589%) |  |
| Class III-V | 6135 (67.035%) | 7311 (78.411%) |  |
| BUN, mg/dL, median (Q1-Q3) | 15.000 (12.000-19.888) | 16.807 (12.325-22.000) | <0.001 |
| Creatinine, mg/dL, median (Q1–Q3) | 0.810 (0.700-0.984) | 0.800 (0.679-0.939) | <0.001 |
| Sodium, mmol/L, mean ± SD | 138.953 ± 3.063 | 138.327 ± 3.340 | <0.001 |
| HCT, %, mean ± SD | 41.030 ± 4.400 | 39.652 ± 5.070 | <0.001 |
| PWR | 33.228 ± 35.337 | 27.044 ± 12.564 | <0.001 |
| 30-day mortality events, n (%) |  |  | <0.001 |
| No | 9047 (98.252%) | 9137 (96.852%) |  |
| Yes | 161 (1.748%) | 297 (3.148%) |  |

ASA, American Society of Anesthesiologists; BUN, blood urea nitrogen; COPD, chronic obstructive pulmonary disease; HCT, hematocrit; PWR, platelet to white blood cell ratio; SD, standard deviation.

TABLE S8 The association between preoperative PWR and 30-day postoperative mortality including all patients with available preoperative PWR values (n = 18031).

| **Exposure** | **Crude model** | | **Model 1** | | **Model 2** | |
| --- | --- | --- | --- | --- | --- | --- |
|  | **HR (95% CI)** | ***P* value** | **HR (95% CI)** | ***P* value** | **HR (95% CI)** | ***P* value** |
| PWR | 0.953 (0.945, 0.962) | <0.0001 | 0.961 (0.952, 0.970) | <0.0001 | 0.974 (0.965, 0.983) | <0.0001 |
| PWR quartile |  |  |  |  |  |  |
| Q1 (0.7-20.1) | 1.00 (Reference) |  | 1.00 (Reference) |  | 1.00 (Reference) |  |
| Q2 (20.1-27.8) | 0.484 (0.383, 0.611) | <0.0001 | 0.521 (0.412, 0.659) | <0.0001 | 0.627 (0.492, 0.799) | 0.0002 |
| Q3 (27.8-37.1) | 0.317 (0.242, 0.416) | <0.0001 | 0.366 (0.278, 0.481) | <0.0001 | 0.493 (0.369, 0.659) | <0.0001 |
| Q4 (37.1-2810.0) | 0.284 (0.214, 0.377) | <0.0001 | 0.350 (0.262, 0.467) | <0.0001 | 0.489 (0.359, 0.665) | <0.0001 |
| *P* for trend | <0.0001 |  | <0.0001 |  | <0.0001 |  |

Model 1: adjusted for age range, sex and race;

Model 2: adjusted for age range, sex, race, severe chronic obstructive pulmonary disease, hypertension, diabetes, systemic infection, open wound infection, disseminated cancer, bleeding disorders, steroid use, emergency case, recent weight loss, functional health status, ventilator, American Society of Anesthesiologists classification, blood urea nitrogen, creatinine, sodium and hematocrit.

CI, confidence interval; HR, hazard ratio; PWR, platelet to white blood cell ratio.

TABLE S9 Threshold effect analysis of preoperative PWR and 30-day postoperative mortality including all patients with available preoperative PWR values (n = 18031).

| **Models** | **HR (95% CI)** | ***P* value** |
| --- | --- | --- |
| Model I |  |  |
| One line effect | 0.974 (0.965, 0.983) | <0.0001 |
| Model II |  |  |
| Turning point (K) | 27.241 |  |
| PWR < K | 0.944 (0.928, 0.960) | <0.0001 |
| PWR ≥ K | 0.998 (0.986, 1.010) | 0.7353 |
| *P* value for LRT test^*^ | <0.001 |  |

Model I, linear analysis; Model II, non-linear analysis.

Adjusted for age range, sex, race, severe chronic obstructive pulmonary disease, hypertension, diabetes, systemic infection, open wound infection, disseminated cancer, bleeding disorders, steroid use, emergency case, recent weight loss, functional health status, ventilator, American Society of Anesthesiologists classification, blood urea nitrogen, creatinine, sodium and hematocrit.

^*^*P* < 0.05 suggests a statistically significant difference between Model II and Model I.

CI, confidence interval; HR, hazard ratio; LRT, log-likelihood ratio test; PWR, platelet to white blood cell ratio.

TABLE S10 The association between preoperative PWR and 30-day postoperative mortality using logistic regression in adult tumor craniotomy.

| **Exposure** | **Crude model** | | **Model 1** | | **Model 2** | |
| --- | --- | --- | --- | --- | --- | --- |
|  | **OR (95% CI)** | ***P* value** | **OR (95% CI)** | ***P* value** | **OR (95% CI)** | ***P* value** |
| PWR | 0.965 (0.954, 0.976) | <0.0001 | 0.971 (0.960, 0.983) | <0.0001 | 0.978 (0.966, 0.990) | 0.0003 |
| PWR quartile |  |  |  |  |  |  |
| Q1 (0.659-18.031) | 1.00 (Reference) |  | 1.00 (Reference) |  | 1.00 (Reference) |  |
| Q2 (18.050-24.674) | 0.501 (0.371, 0.677) | <0.0001 | 0.538 (0.397, 0.729) | <0.0001 | 0.576 (0.416, 0.796) | 0.0008 |
| Q3 (24.678-33.455) | 0.371 (0.267, 0.517) | <0.0001 | 0.406 (0.290, 0.567) | <0.0001 | 0.498 (0.349, 0.711) | 0.0001 |
| Q4 (33.457-100.000) | 0.371 (0.267, 0.517) | <0.0001 | 0.445 (0.317, 0.625) | <0.0001 | 0.502 (0.344, 0.733) | 0.0004 |
| *P* for trend | <0.0001 |  | <0.0001 |  | 0.0002 |  |

Model 1: adjusted for age range, sex and race;

Model 2: adjusted for age range, sex, race, severe chronic obstructive pulmonary disease, hypertension, diabetes, systemic infection, open wound infection, disseminated cancer, bleeding disorders, steroid use, emergency case, recent weight loss, functional health status, ventilator, American Society of Anesthesiologists classification, blood urea nitrogen, creatinine, sodium and hematocrit.

CI, confidence interval; OR, odds ratio; PWR, platelet to white blood cell ratio.

TABLE S11 Threshold effect analysis of preoperative PWR and 30-day postoperative mortality using logistic regression in adult tumor craniotomy.

| **Models** | **OR (95% CI)** | ***P* value** |
| --- | --- | --- |
| Model I |  |  |
| One line effect | 0.978 (0.966, 0.990) | 0.0003 |
| Model II |  |  |
| Turning point (K) | 20.000 |  |
| PWR < K | 0.914 (0.883, 0.945) | <0.0001 |
| PWR ≥ K | 0.997 (0.984, 1.011) | 0.7033 |
| *P* value for LRT test^*^ | <0.001 |  |

Model I, linear analysis; Model II, non-linear analysis.

Adjusted for age range, sex, race, severe chronic obstructive pulmonary disease, hypertension, diabetes, systemic infection, open wound infection, disseminated cancer, bleeding disorders, steroid use, emergency case, recent weight loss, functional health status, ventilator, American Society of Anesthesiologists classification, blood urea nitrogen, creatinine, sodium and hematocrit.

^*^*P* < 0.05 suggests a statistically significant difference between Model II and Model I.

CI, confidence interval; OR, odds ratio; LRT, log-likelihood ratio test; PWR, platelet to white blood cell ratio.
